# Postoperative liver dysfunction in gastric cancer patients following early enhanced nutritional protocols: a retrospective observational study

**DOI:** 10.1186/s40780-025-00473-y

**Published:** 2025-08-04

**Authors:** Kazuo Kobayashi, Satoshi Ida, Masanari Tsuji, Arisa Nawa, Takeshi Aoyama, Takashi Yokokawa, Yoshikazu Tateai, Shuichi Watabe, Hisanori Shimizu, Souya Nunobe, Masakazu Yamaguchi

**Affiliations:** 1 Department of Pharmacy, Cancer Institute Hospital, Japanese Foundation for Cancer Research, Koto-Ku, 3-8-31 Ariake Koto-Ku, ResearchTokyo, 135-8550 Japan; 2 Department of Gastroenterological Surgery, Cancer Institute Hospital, Japanese Foundation for Cancer Research, Koto-Ku, Tokyo, Japan; 3 Ain Pharmaciez Inc., Sapporo, Hokkaido Japan; 4 Department of Clinical Pharmacology, School of Pharmacy, Tokyo University of Pharmacy and Life Sciences, Hachioji, Tokyo, Japan

**Keywords:** Gastric cancer, Postoperative nutritional management, Liver dysfunction, Acetaminophen

## Abstract

**Background:**

Postoperative liver dysfunction has been　recognized as a potential complication following gastric cancer surgery, particularly in the context of enhanced perioperative nutritional management. Although early supplementation with amino acids and fat emulsions has been introduced to optimize recovery, its impact on postoperative liver function remains insufficiently elucidated. This study aimed to evaluate the incidence, time course, and risk factors of postoperative liver dysfunction, and to assess the safety of early nutritional support in this context.

**Methods:**

This retrospective observational study included patients who underwent radical gastrectomy between January and July 2020 at Cancer Institute Hospital Ariake. All patients received early postoperative nutritional support including amino acid and fat emulsion supplementation. Liver function was assessed based on serum aspartate aminotransferase (AST), alanine aminotransferase (ALT), and total bilirubin (T-Bil) levels on postoperative days (POD) 1, 3, 7, as well as at the first outpatient visit. Postoperative liver dysfunction was defined according to the Common Terminology Criteria for Adverse Events (CTCAE) version 5.0. Risk factors for liver dysfunction on POD 7 were assessed using by univariate and multivariate analyses.

**Results:**

Data for 170 patients were analyzed. AST and ALT levels showed a biphasic pattern, with two distinct peaks observed on POD 1 and POD 7. The incidence rates of Grade 1 or higher elevations were 51.8% for AST and 39.4% for ALT on POD 1, and 47.9% for AST and 42.0% for ALT on POD 7. At the first outpatient visit, the incidence declined markedly. Univariate analysis identified acetaminophen use as a significant risk factor for liver dysfunction on POD 7 (*p* < 0.05), with open surgery and extensive procedures showing trend-level associations. Multivariate analysis confirmed acetaminophen use as an independent risk factor. Importantly, no significant association was found between liver dysfunction and the administration of amino acids or fat emulsions, suggesting that early postoperative nutritional management appears to be safe.

**Conclusions:**

Postoperative liver dysfunction is not uncommon in gastric cancer patients but is generally transient and clinically manageable. Early postoperative nutritional management, including amino acid and fat emulsion supplementation, appears to be safe and does not adversely affect postoperative liver function when appropriately administered. Careful monitoring of liver function, particularly in patients receiving acetaminophen, remains essential to optimize postoperative outcomes.

## Introduction

Gastric cancer is the fifth most common malignancy worldwide and the third leading cause of cancer-related mortality, with an estimated 1.09 million new cases and 769,000 deaths reported in 2020 [1]. Gastrectomy is the standard treatment for resectable gastric cancer [2]. To promote postoperative recovery in these patients, enhanced recovery after surgery (ERAS) protocols have been implemented in many centers to improve postoperative recovery for gastric cancer patients, demonstrating favorable outcomes [3]. Among the various components of ERAS, perioperative nutritional management plays a particularly critical role. As part of the postoperative recovery enhancement protocol implemented at our institution, As part of the postoperative recovery enhancement protocol implemented at our institution, early postoperative administration of the three major nutrients and fat emulsions has been introduced, targeting an energy intake of approximately 1,500 kcal/day [4, 5].

Meanwhile, postoperative hepatic dysfunction has been recognized as a potential complication following gastric cancer surgery [6, 7].Several perioperative risk factors for liver dysfunction have been identified, including advanced age, malnutrition, pre-existing liver disease, and the use of certain medications [8]. However, the impact of early postoperative nutritional interventions on liver function in gastric cancer patients remains insufficiently elucidated.

In evaluating liver dysfunction, aspartate aminotransferase (AST) and alanine aminotransferase (ALT) are widely accepted and sensitive biomarkers of hepatocellular injury. These enzymes respond early to metabolic and drug-related hepatic stress, making them suitable for assessing transient liver dysfunction in the immediate postoperative period.

Therefore, in this study, we aim to investigate the frequency and timing of postoperative liver dysfunction in gastric cancer patients and to identify potential perioperative risk factors associated with its development. Particular attention was paid to AST and ALT trends over time, and we focused on evaluating whether early postoperative supplementation with amino acids and fat emulsions is safe and does not contribute to liver dysfunction.

## Methods

### Study design and eligible patients

This retrospective observational study was conducted to investigate the frequency, timing, and risk factors for postoperative liver dysfunction in patients undergoing gastric cancer surgery. Patients who underwent radical gastrectomy between January 1, 2020, and July 31, 2020, at Cancer Institute Hospital Ariake were included. From postoperative day (POD) 1, a combination of BFLUID® infusion, which contains vitamin B1, glucose, electrolytes, and amino acids, and 20% Intralipos® lipid emulsion (250 mL) was administered. Additionally, oral intake was initiated on postoperative day 1 with fluid intake, progressing to a semi-liquid diet (three-quarters porridge) by postoperative day 3. Subsequently, the diet advanced daily to five-tenths porridge and then full porridge, facilitating early oral intake. Furthermore, from postoperative day 3, jelly processed from the elemental diet (Elental® powder, 250 kcal, twice daily) was added as a snack, resulting in an average daily total caloric intake of 1,577 kcal. For postoperative pain management, acetaminophen tablets or Acetlio® intravenous infusion was administered as needed.

BFLUID® infusion was administered at a rate of approximately 83.3 mL/h over 24 h (total volume 2000 mL/day), delivering a daily amino acid dose of approximately 60 g. Intralipos® 20% (250 mL) was administered intravenously over approximately 5 h (approximately 50 mL/h) once daily from POD 1 to POD 5.

Acetaminophen was used as needed for postoperative pain control. Either oral acetaminophen (500 mg/tablet) or intravenous Acelio® (1000 mg/100 mL) was given depending on the clinical situation, with reference to pain scores on the numerical rating scale (NRS), typically when the score was around 4 or higher.

The oral intake protocol was standardized for all patients. Fluid intake began on POD 1, progressing to semi-liquid diet (three-quarters porridge) by POD 3, followed by five-tenths and then full porridge. Deviations from the protocol were permitted only in cases of postoperative nausea, ileus, or aspiration risk.

### Endpoints

The primary endpoints of this study were (1) to identify perioperative risk factors associated with postoperative liver dysfunction and (2) to characterize the timing and frequency of postoperative liver dysfunction in gastric cancer patients.

Liver dysfunction was assessed using serum levels of total bilirubin (T-Bil), AST, and ALT as per the CTCAE version 5.0.The reference ranges were as follows:

- T-Bil: ≤ 1.5 mg/dL for both males and females.
- AST: ≤ 30 IU/L for males and ≤ 35 IU/L for females.
- ALT: ≤ 42 IU/L for males and ≤ 23 IU/L for females. Liver dysfunction was defined as AST or ALT grade 1 or higher.

The observation period for this study began on the day immediately before surgery and continued until the day of the first outpatient visit after discharge from the hospital, approximately 1 month after surgery. As a secondary endpoint, we evaluated the safety of early postoperative nutritional interventions, particularly the administration of amino acids and fat emulsions, by examining their association with liver dysfunction. In addition, we calculated the AST/ALT ratio on POD 1 and POD 7 to assess the hepatic origin of transaminase elevations. The ratio was calculated using routinely collected AST and ALT values at each time point. This analysis was intended to provide further insights into the etiology of observed enzyme elevation patterns.

### Data collection

Clinical and hematological data were collected and retrospectively examined using electronic medical records. Blood samples were routinely obtained on the day before surgery, and on POD 1, 3, 7, and at the first outpatient visit (typically between POD 10 and 14). Blood tests were performed at all time points unless contraindicated or missed due to early discharge or other clinical reasons. Missing laboratory data were excluded from the respective analyses and were not imputed. Serum albumin levels were collected from preoperative blood tests conducted within 7 days prior to surgery. Based on the institutional reference standard at Cancer Institute Hospital Ariake, hypoalbuminemia was defined as serum albumin < 4.1 mg/dL.The severity of postoperative complications was graded according to the Clavien-Dindo classification [9]. Postoperative complications were defined as those of Clavien-Dindo grade II or above, occurring within 30 days of surgery. This definition was chosen because Grade II or higher complications require pharmacological treatment or more invasive interventions, which have greater clinical relevance and prognostic significance compared to Grade I events. Additionally, this threshold has been widely used in prior surgical studies [10], facilitating comparability and interpretability of the results.

### Statistical analysis

Preoperative albumin levels of less than 4.1 mg/dL, which is the institutional standard value at Cancer Institute Hospital Ariake, were used for statistical analysis. Univariate and multivariate analyses were performed to identify risk factors for hepatic dysfunction. The χ2 test or Fisher's exact probability test was used for univariate analysis. Multiple logistic regression analysis was employed for multivariate analysis to identify independent risk factors for liver dysfunction on POD 7. Among liver enzyme parameters, multivariate analysis was performed using AST elevation as the dependent variable, based on its clinical significance and observed statistical trends. Variables were selected post hoc based on clinical relevance, biological plausibility, and previous reports suggesting potential associations. The variables selected for multivariate analysis were based on their clinical relevance and previously reported associations with postoperative liver dysfunction, as described in prior studies [6, 7]. The final model included acetaminophen use, surgical approach (laparotomy vs. laparoscopy), surgical procedure (total vs. distal gastrectomy), and presence of preoperative liver disease. Presence of preoperative liver disease was included as an independent variable to account for baseline liver function abnormalities. To assess for multicollinearity among explanatory variables, we calculated the variance inflation factor (VIF). All VIF values were below 2.0, indicating no significant multicollinearity. The significance level was set at *p* < 0.05. Statistical analyses were conducted using IBM SPSS version 24.0 (SPSS (Co., Ltd.), Tokyo).

## Results

### Study population

**Fig. 1:**
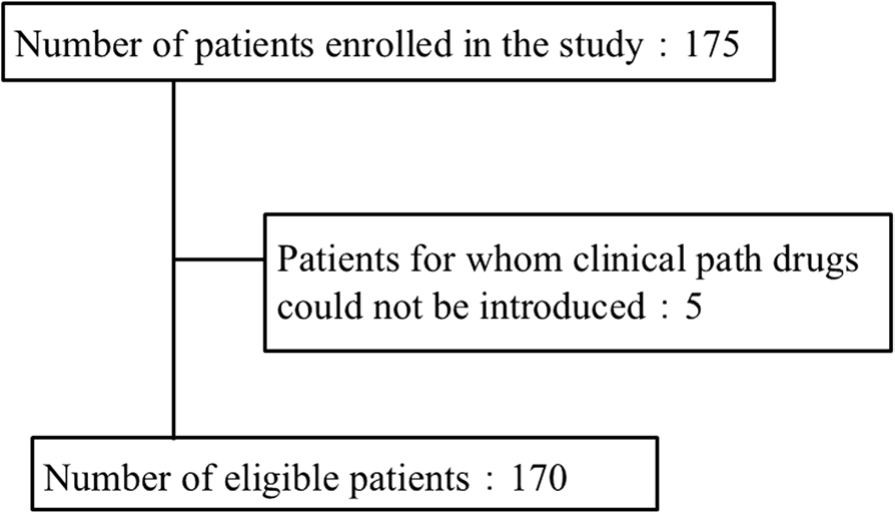
Flow diagram of the study design

Figure 1 presents the study population. Among 175 patients who underwent radical gastrectomy during the study period, 170 patients who were introduced to clinical path agents were included in the analysis. Meanwhile, 5 patients who were not introduced to clinical path agents, such as fat emulsion, were excluded from the study.

### Patient characteristics

Patient characteristics are summarized in Table 1. Laparoscopic surgery was performed in 79.4% (135/170).

**Table 1:** Patient characteristics

Characteristics	*N* = 170
Gender Male/female	98 / 72
Age (years)^a^	68 (29–87)
Preoperative body weight (kg)^a^	61 (35–123)
Clinical Stage I /II / III / IV	103 / 36 / 24 / 7
Surgical approach: Open / Laparoscopic	35 / 135
Surgical procedure	
Distal gastrectomy	95
Pylorus-preserving gastrectomy	19
Proximal gastrectomy	19
Total gastrectomy	37
Preoperative laboratory values^a^	
Albumin (g/dL)	4.1 (2.4–5.0)
Total bilirubin (mg/dL)	0.6 (0.2–1.7)
γ-GTP (IU/L)	23 (6–192)
AST (IU/L)	22 (9–64)
ALT (IU/L)	18 (7–85)
LDH (IU/L)	180 (114–356)
BUN (mg/dL)	15 (6–27)
S-CRE (mg/dL)	0.73 (0.38–1.46)
CRP (mg/dL)	0.06 (0.01–2.92)
PreALB (mg/dL)	25.9 (12.7–50.9)

*AST* Aspartate aminotransferase, *ALT* Alanine aminotransferase, *LDH* Lactate dehydrogenase, *BUN* Blood urea nitrogen, *S-CRE* Serum creatinine, *CRP* C-reactive protein, *PreALB* Prealbumin

^a^median (range)

Highly invasive procedures, including total gastrectomy and proximal gastrectomy, made up 32.9% (56/170) of all surgeries, with 37 total gastrectomies and 19 proximal gastrectomies performed. Median preoperative liver function was within normal range.

### Trend of AST and ALT levels

Although the total number of patients was 170, laboratory data were not available for one patient on POD 7 and for six patients at the first outpatient visit. Therefore, the analyses were conducted with 169 patients on POD 7 and 164 patients at the outpatient visit, respectively.

**Fig. 2:**
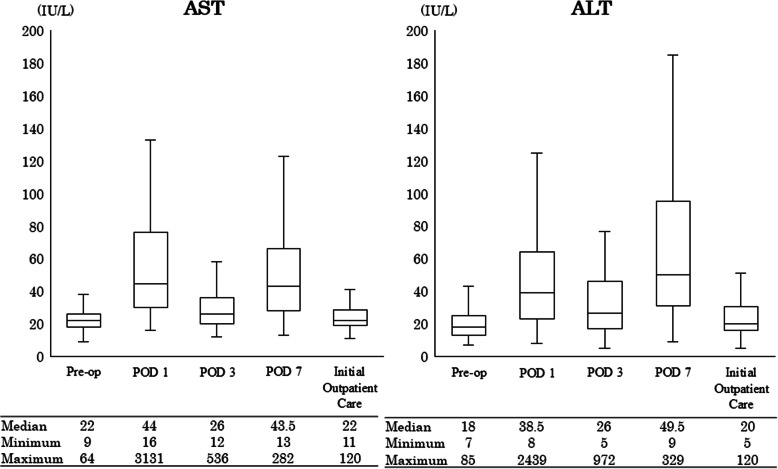
Changes in AST and ALT before and after surgery. Values are expressed as medians (horizontal line in box) and interquartile ranges (25th–75th percentiles; box limits). Whiskers represent the minimum and maximum values excluding outliers. Sample size (n) is indicated for each time point. Pre-op: Pre-operation, POD: postoperative day

**Fig. 3:**
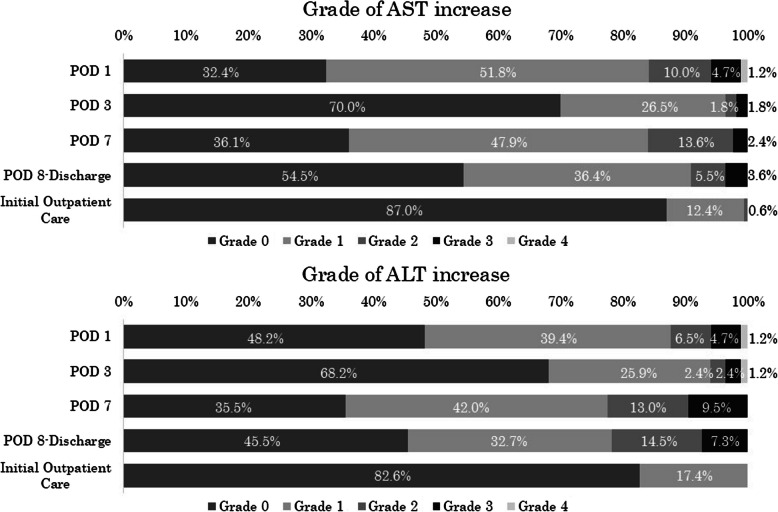
Incidence of increased AST and ALT by Grade. Pre-op: Pre-operation, POD: postoperative day

**Fig. 4:**
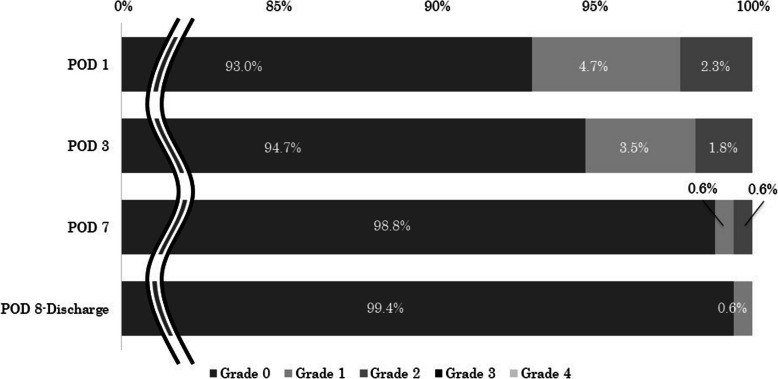
Incidence of increased blood bilirubin by Grade. POD: postoperative day

Figure 2 graphically illustrates the changes in AST and ALT levels before and after surgery. Both AST and ALT exhibited biphasic peaks on the first and seventh postoperative days. Figure 3 presents the incidence of increased AST and ALT levels by grade. The incidence of Grade 1 or higher increases in AST and ALT was 51.8% and 39.4%, respectively, on POD 1, 47.9% and 42.0%, respectively, on POD 7. In contrast, at the first outpatient visit, the incidence was only 0.6% for AST and 0% for ALT. In addition, the incidence of Grade 2 or higher increases in T-bil was 2.3% on POD 1, 0.6% on POD 7, and 0% at the first outpatient visit (Fig. 4). The AST/ALT ratio was calculated on POD 1 and POD 7. The median (IQR) was 1.15 (0.97–1.53) on POD 1 and 0.91 (0.72–1.15) on POD 7, indicating a decreasing trend over time.

### Risk factors for liver dysfunction on postoperative day 7

Risk factors for liver dysfunction on POD 7 are analyzed and presented in Table 2. The fat emulsion administration rate exceeded the recommended guideline of 0.1 g/kg/hr for enteral feeding. By univariate analysis, the use of acetaminophen was found to be a significant risk factor (*p* < 0.05). Multivariate analysis revealed that only acetaminophen use was an independent risk factor for liver dysfunction on POD 7.

**Table 2:** Risk factors for the development of Grade ≥ 1 AST elevation on POD 7 (AST-based liver dysfunction)

	**Univariate analysis**		**Multivariate analysis**	
	OR (95%Cl)	*p*-value	OR (95%Cl)	*p*-value
Gender (Male)	1.37 (0.72–2.58)	0.337		
Age (≥ 65)	1.16 (0.61–2.21)	0.744		
Fat emulsion dosing rate (> 0.1 g/kg/hr)	0.73 (0.26–2.01)	0.627	0.87 (0.30–2.45)	0.793
Use of acetaminophen (Yes)	2.48 (1.30–4.71)	0.006	2.54 (1.32–4.90)	0.005
Laparotomy (Yes)	1.66 (0.78–3.54)	0.183	1.53 (0.65–3.60)	0.325
Total gastrectomy/ Proximal gastrectomy (Yes)	1.72 (0.89–3.33)	0.103	1.55 (0.75–3.22)	0.231
Preoperative liver disease complications (yes)	2.30 (0.25–21.12)	0.405	2.55 (0.24–27.09)	0.436
Preoperative ALB level (< 4.1 mg/dL)	0.60 (0.31–1.12)	0.112		
Postoperative complications (Yes)	1.95 (0.60–6.26)	0.255		

Variables included in the multivariate model were selected based on clinical relevance and prior literature, not on univariate *p*-values

*ALB* Albumin, *CI* Confidence interval, *OR* Odds ratio

## Discussion

This retrospective observational study investigated the frequency, timing, and risk factors for postoperative liver dysfunction in gastric cancer patients. Among 170 patients undergoing radical gastrectomy, transient elevations in AST and ALT were observed on postoperative days (POD) 1 and 7. The early peak likely reflects surgical stress [6], while the later peak may be associated with perioperative medication use, with a particular association observed for acetaminophen administration [8]. These findings provide real-world complementary insights into the temporal characteristics of postoperative liver enzyme fluctuations.

Previous studies have reported postoperative liver dysfunction, especially in laparoscopic procedures. For example, Etoh et al. suggested that CO₂ pneumoperitoneum could reduce hepatic blood flow, contributing to transient liver enzyme elevations [7]. Jeong et al. also identified multifactorial contributors such as liver manipulation and arterial ligation [6]. However, our study did not focus on laparoscopic-specific effects, and instead identified acetaminophen use and extensive surgery as significant or trend-level risk factors. These results emphasize the multifactorial nature of liver dysfunction after gastric cancer surgery.

In the present study, liver dysfunction was defined as Grade 1 or higher elevations in AST or ALT according to CTCAE version 5.0. While previous studies [11, 12] have defined postoperative liver dysfunction using stricter criteria—Grade ≥ 3 or Grade ≥ 2, respectively—our definition was intentionally broad to enhance sensitivity in detecting subtle hepatic alterations.

This approach allowed for the comprehensive assessment of early and transient enzyme elevations that may reflect subclinical liver stress, especially in the context of enhanced nutritional protocols. Importantly, our primary goal was not to identify severe hepatic injury but rather to detect early biochemical changes and their potential risk factors.

Moreover, the clinical relevance of Grade 1 enzyme elevations should not be underestimated, as such elevations may serve as early warning signs in patients receiving multiple perioperative interventions, including acetaminophen or lipid-based infusions. Although liver dysfunction was broadly defined to include elevations in either AST or ALT, only AST elevation demonstrated significant associations in univariate analysis. Therefore, AST was selected as a representative endpoint for the multivariate model.

Among AST and ALT, only AST elevation on POD 7 was significantly associated with perioperative factors such as acetaminophen use in univariate analysis. Therefore, multivariate analysis was conducted focusing on AST as the outcome of interest, to maintain model parsimony and avoid overadjustment based on non-significant variables. While ALT was also monitored, it did not show significant associations and was excluded from further modeling. As detailed information on acetaminophen dosage and administration timing was not collected, the observed association should be interpreted with caution and does not imply causality. In order to further interpret the etiology of transaminase elevations, we analyzed the AST/ALT ratio. Since AST is not specific to the liver and can be elevated due to muscle injury or hemolysis, the AST/ALT ratio can help infer the likely source of enzyme elevation. In our cohort, the ratio decreased from POD 1 to POD 7. The higher ratio observed on POD 1 may reflect a broader source of AST, such as surgical stress or muscle damage, while the lower ratio on POD 7 suggests a shift toward liver-specific enzyme release. This finding reinforces the hypothesis that late-onset transaminase elevation may reflect hepatic stress potentially associated with pharmacological or nutritional interventions.

Nevertheless, to ensure comparability with prior studies, we also conducted stratified analyses by CTCAE grade and confirmed that the majority of severe cases (Grade ≥ 3) were rare, supporting the generally mild and transient nature of the observed dysfunctions.

Importantly, early postoperative nutritional management using fat emulsions and amino acid solutions was not associated with liver dysfunction. Although previous reports have raised concerns about soybean oil–based fat emulsions potentially exacerbating inflammation and immune suppression [13], adherence to proper administration rates (≤ 0.1 g/kg/hr) appeared to mitigate such risks. Similarly, theoretical concerns regarding hepatic strain from amino acid supplementation were not supported by our data, consistent with previous guidance on individualized parenteral nutrition [14].

These findings suggest that early postoperative nutritional support using fat emulsions and amino acid solutions is safe when administered at appropriate doses and infusion rates, consistent with recommended protocols. The results also highlight the need for careful selection and monitoring of perioperative medications, especially acetaminophen [8], to reduce the risk of liver dysfunction.

This study has several limitations. As a single-center retrospective analysis, its generalizability may be limited. In addition, long-term liver outcomes beyond the first outpatient visit were not assessed. Moreover, time-course data on perioperative drug administration—including acetaminophen and antibiotics—were not consistently available. Potential variability in actual infusion rates and adherence to nutritional protocols, while minimized through standardized clinical pathways, cannot be fully excluded. Lastly, while we did not comprehensively assess other possible causes of liver dysfunction—such as infection, antibiotic-induced liver injury, or other postoperative complications including gastrointestinal symptoms—we acknowledge that these unmeasured factors may have influenced the observed outcomes. Moreover, the absence of a control group precludes definitive conclusions about the overall safety of early nutritional intervention. Therefore, its safety should be interpreted with caution and confirmed in future controlled prospective studies. These limitations, including the lack of data on liver enzyme normalization, hepatoprotective agent use, and discharge delays, should be addressed in future prospective studies. The dataset used in this study was collected in early 2020. While clinical practices may have evolved since then, the surgical procedures, perioperative management protocols, and nutritional interventions applied during this period remain largely consistent with current standard practices at our institution. Therefore, the findings remain relevant to present-day clinical care. Detailed data on daily caloric and nitrogen intake per body weight were not consistently available and were therefore not analyzed. Future prospective studies should incorporate this information for more precise evaluation.

In conclusion, postoperative liver dysfunction is relatively common in gastric cancer patients but generally transient and manageable. Early nutritional support with amino acids and fat emulsions appears to be safe with respect to liver function when administered appropriately; nonetheless, further controlled studies are needed to comprehensively evaluate overall safety, including other postoperative complications. Acetaminophen use, in particular, remains a significant risk factor, particularly around POD 7. Further prospective studies are warranted to validate these findings and optimize perioperative liver function management strategies.

## Data Availability

Data supporting the findings of this study are available from the corresponding author upon reasonable request. The data are not publicly available due to privacy and ethical restrictions.

## Abbreviations

ALT: Alanine Aminotransferase
AST: Aspartate Aminotransferase
BMI: Body Mass Index
CTCAE: Common Terminology Criteria for Adverse Events
IRB: Institutional Review Board
POD: Postoperative Day
T-Bil: Total Bilirubin
